# Comparison of Antioxidative Effects of Insect Tea and Its Raw Tea (Kuding Tea) Polyphenols in Kunming Mice

**DOI:** 10.3390/molecules23010204

**Published:** 2018-01-19

**Authors:** Xin Zhao, Jia-Le Song, Ruokun Yi, Guijie Li, Peng Sun, Kun-Young Park, Huayi Suo

**Affiliations:** 1 Chongqing Collaborative Innovation Center for Functional Food, Chongqing University of Education, Chongqing 400067, China; zhaoxin@cque.edu.cn (X.Z.); yirk@cque.edu.cn (R.Y.); quajetlee@gmail.com (G.L.); sunpeng@foods.ac.cn (P.S.); 2 Chongqing Engineering Research Center of Functional Food, Chongqing University of Education, Chongqing 400067, China; 3 Chongqing Engineering Laboratory for Research and Development of Functional Food, Chongqing University of Education, Chongqing 400067, China; 4 College of Biological and Chemical Engineering, Chongqing University of Education, Chongqing 400067, China; 5 Department of Nutrition and Food Hygiene, School of Public Health, Guilin Medical University, Guilin 541004, Guangxi, China; songjiale@glmc.edu.cn; 6 Department of Food Science and Biotechnology, Cha University, Seongnam 13488, Gyeongghi-do, Korea; 7 College of Food Science, Southwest University, Chongqing 400715, China

**Keywords:** insect tea, Kuding tea, polyphenol, antioxidation, mice

## Abstract

Kudingcha is a traditional Chinese tea, and insect tea is a special drink produced by the metabolism of insect larvae using the raw Kuding tea. Insect tea polyphenols (ITP) and its raw tea (Kuding tea) polyphenols (KTP) are high-purity polyphenols extracted by centrifuge precipitation. The present study was designed to compare the antioxidative effects of insect tea polyphenols (ITP) and its raw tea (Kuding tea) polyphenols (KTP) on d-galactose-induced oxidation in Kunming (KM) mice. KM mice were treated with ITP (200 mg/kg) and KTP (200 mg/kg) by gavage, and vitamin C (VC, 200 mg/kg) was also used as a positive control by gavage. After determination in serum, liver and spleen, ITP-treated mice showed higher superoxide dismutase (SOD), glutathione peroxidase (GSH-Px), and glutathione (GSH) activities and lower nitric oxide (NO), malonaldehyde (MDA) activities than VC-treated mice, KTP-treated mice and untreated oxidation mice (control group). By H&E section observation, the mice induced by d-galactose-induced oxidation showed more changes than normal mice, and oxidative damage appeared in liver and spleen tissues; ITP, VC and KTP improved oxidative damage of liver and spleen tissues, and the effects of ITP were better than VC and KTP. Using quantitative polymerase chain reaction (qPCR) and western blot experiments, it was observed that ITP could increase the mRNA and protein expression of neuronal nitric oxide synthase (nNOS), endothelial nitric oxide synthase (eNOS), manganese superoxide dismutase (Mn-SOD), cupro/zinc superoxide dismutase (Cu/Zn-SOD), catalase (CAT), heme oxygenase-1 (HO-1), nuclear factor erythroid 2 related factor 2 (Nrf2), gamma glutamylcysteine synthetase (γ-GCS), and NAD(P)H:quinone oxidoreductase 1 (NQO1) and reduce inducible nitric oxide synthase (iNOS) expression in liver and spleen tissues compared to the control group. These effects were stronger than for VC and KTP. Both ITP and KTP had good antioxidative effects, and after the transformation of insects, the effects of ITP were better than that of KTP and even better than VC. Thus, ITP can be used as an antioxidant and anti-ageing functional food.

## 1. Introduction

Ageing is a spontaneous biological process wherein the biological body gradually shows a decline in function, adaptability and immunity. Ageing is closely associated with many diseases, including hypertension, type 2 diabetes, atherosclerosis and Alzheimer’s disease. The onset of these diseases can be slowed by delaying ageing [1]. d-galactose is recognized as an agent that can control ageing. The ageing animal model used for replication is similar to natural ageing and has been widely used in the model construction of aged animals [2]. A small amount of normal body d-galactose can be transformed into glucose and involved in metabolism, but a large amount of d-galactose will lead to cellular metabolic disorders and alter activity of tissue cell oxidase to produce a large number of superoxide anion and oxidation products; the latter process will lead to biological macromolecular structure and functional damage, eventually leading to cell ageing [3]. Antioxidant components in natural plants have been gradually applied to the development of antioxidant and health care products after the antioxidant effect was verified by the d-galactose model test [4].

Insect tea is a special solid brewing tea with a peculiar smell and rich characteristics that is popular among Chinese minorities. It is mainly produced in remote areas in southwestern China. Locals collect wild broadleaf holly, incense leaves and other fresh leaves from wild plants. They cook them to remove the astringent taste and the naturally dried leaves are stacked into the layers of casks, each layer evenly topped with scouring rice water. After some time, the leaves are slightly naturally fermented and emit an aroma that attracts Hydrillodes repugnalis and other insects to lay eggs on the leaves. The hatching larvae feed on leaves, excluding insect faces particles. Locals select larvae facal particles, remove miscellaneous leaves and produce the insect tea after exposure to frying and other techniques [5]. The nutritional value of insect tea is higher than that of common tea, containing a variety of amino acids, crude protein, crude fat, carbohydrate, vitamins and other nutrients, but it also contains trace elements needed by the body, especially abundant polyphenols [6]. Kuding tea also contains polyphenols, including chlorogenic acid, cryptochlorogenic acid, and isochlorogenic acids A, B, and C (unpublished work by our team). As an important raw tea for insect tea, Kuding tea is also a widely consumed health tea in southern China, especially in the important producing area for insect tea, Guizhou province [7]. Studies have shown that Kuding tea has a variety of effects, including antioxidation, anti-inflammatory and anti-cancer activity [8,9]. Kuding tea is rich in polyphenols, which have a strong ability to scavenge free radicals. It can increase enzyme activity in the body by blocking lipid peroxidation, which plays a role in anti-mutation and anti-cancer effects. study indicates that polyphenols in Kuding tea play a very important role in their functional effect [10]. In recent years, studies have found that insect tea contains a variety of functional ingredients, including polyphenols, mineral elements and amino acids, of which the content of polyphenols is close to 10%; these studies have shown that Kuding tea as a raw material and after metabolism of insect tea still retains a high content of polyphenols; the health benefits of insect tea may also be related to its high content of polyphenols [10,11,12]. 

Tea polyphenols are a very important functional component in tea. Studies have shown that tea polyphenols have many physiological functions, including antioxidant capabilities, for example in the prevention of cardiovascular diseases, and anti-tumor, anti-radiation, antibacterial and anti-virus, anti-inflammatory activities [13,14,15]. Studies have also shown that the antioxidation and scavenging free radicals of tea polyphenols are mainly due to the structure of catechol in tea polyphenols, which has a strong metal ion chelation ability, thus reducing the catalytic function of metal ions on the oxidation reaction. Tea polyphenols can scavenge superoxide anion free radicals and hydroxyl free radicals, thereby reducing oxidative damage to cellular DNA and other molecules. At the same time, tea polyphenols can inhibit oxidase and reduce the generation of free radicals by quenching free radicals, enhancing antioxidant enzyme activity, such as enhancing glutathione peroxidase, catalase, ester reductase, glutathione-*S*-transferase activity; in addition, tea polyphenols can also react with peroxyl radicals, thereby terminating the lipid peroxidation chain reaction [16,17,18].

This study established an in vivo animal oxidation model so that the antioxidative effect of insect tea polyphenols can be scientifically determined using molecular biological methods, as well as the enhancement of antioxidant effects of polyphenols from raw tea (Kuding tea) by biochemical effects in insects. d-galactose can be converted into aldoses and hydrogen peroxide in vivo, causing oxidative damage to many types of large biological molecules and causing cell oxidation and senescence in mammals. It has been widely applied in the study of antioxidant mechanisms and screening of antioxidant components [19]. Oxidation-related enzymes, such as SOD, GSH, GSH-Px, and CAT, are all apparent in the oxidation process. NOS is also present under the oxidation state. This animal model and oxidative stress biomarkers were chosen to study antioxidation. Insect tea has appeared on the market as a commodity, but its functional effect is still lacking scientific research. To evaluate the antioxidant effects of insect tea polyphenols scientifically, this study chose samples of China Guizhou Insect tea and its raw tea (Kuding tea) polyphenols as the research object for the experiment. Insect tea samples and raw tea (Kuding tea) polyphenol antioxidant effects were compared, and the data obtained will help further study the functional role of insect tea and the role of insects on plants, enhancing the effects of insects on plant leaves.

## 2. Results

### 2.1. Contents of Polyphenols

**Figure 1:**
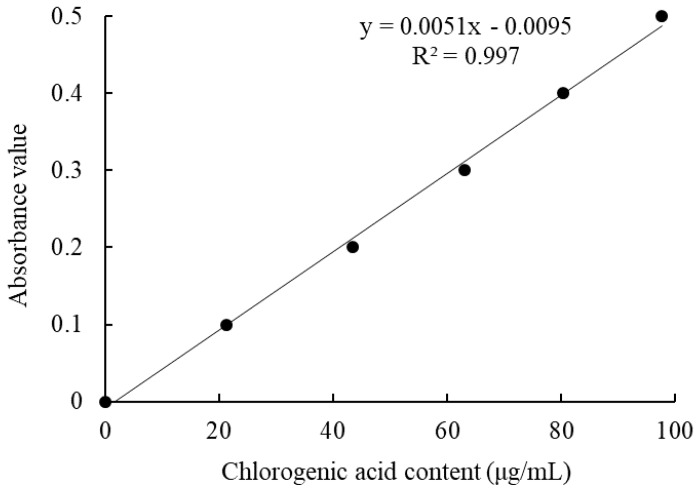
Standard curve of chlorogenic acid.

Based on the determination of the chlorogenic acid standard absorbance curve with equation of the line Y = 0.0051X − 0.0095 (R^2^ = 0.997, Y is the absorbance value, X is the chlorogenic acid content) (Figure 1 and Table 1), the polyphenol contents of KTP and ITP were calculated from the standard curve as 71.38% and 69.82%. The polyphenols contents of KTP and ITP were adjusted to the same concentration at 200 mg/kg body weight for the animal experiments.

### 2.2. Activities of NO, SOD, GSH-Px, GSH and MDA in Mice

As shown in Table 2, Table 3 and Table 4, the SOD, GSH-Px, GSH activities in the serum, liver and spleen of the mice in normal group were highest in all groups; the activities in the control group were the lowest. The NO and MDA activities of mice in the control group were highest, but the activities in normal group were lowest. The ITP-treated mice had higher SOD, GSH-Px, GSH activities and lower NO, MDA activities than those of VC- and KTP-treated mice. 

### 2.3. H&E Pathological Observation

**Figure 2:**
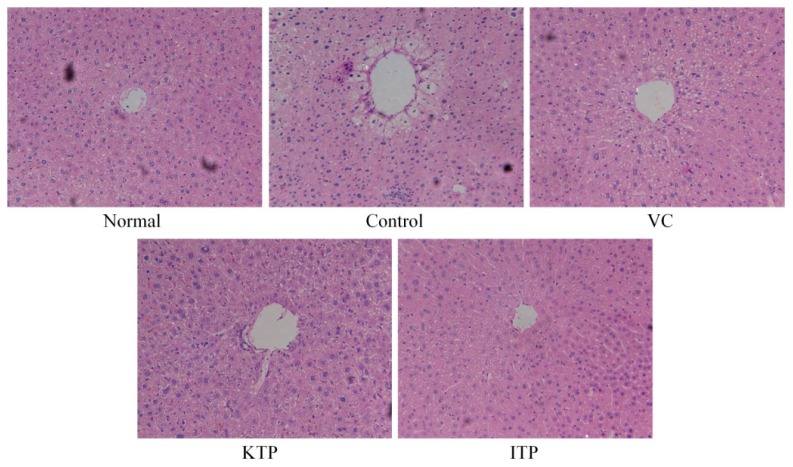
H&E pathological observation of liver in mice. Magnification 100×. VC: vitamin C (200 mg/kg body weight); KTP: Kuding tea polyphenols (200 mg/kg body weight); ITP: insect tea polyphenols (200 mg/kg body weight).

As shown in Figure 2, the hepatic lobules and stem cells of the hepatic tissue in normal group mice were normal; the hepatic cord was arranged regularly, the hepatic sinusoids were normal, and the central vein was also normal. The volume of the hepatocytes of hepatic tissue in control group mice was narrowed; the volume of the nucleus was also reduced, and the staining was enhanced; the liver cells were loosely connected, and liver cells showed fatty degeneration. There were different sizes of vacuoles, hepatic disorders, hepatic sinusoidal distortion, stenosis and even occlusion in the degenerative hepatic cytoplasm. The hepatocytes of ITP, VC and KTP were larger than for mice in the control group, and the nuclei were large; a small amount of cytoplasm for the hepatocytes were seen in the lipid droplets; the hepatocyte cords were also wider than in mice in the control group. The hepatic tissue morphology of ITP-treated mice was closest to mice in the control group, and the morphology of VC-treated mice was better than that of KTP-treated mice.

**Figure 3:**
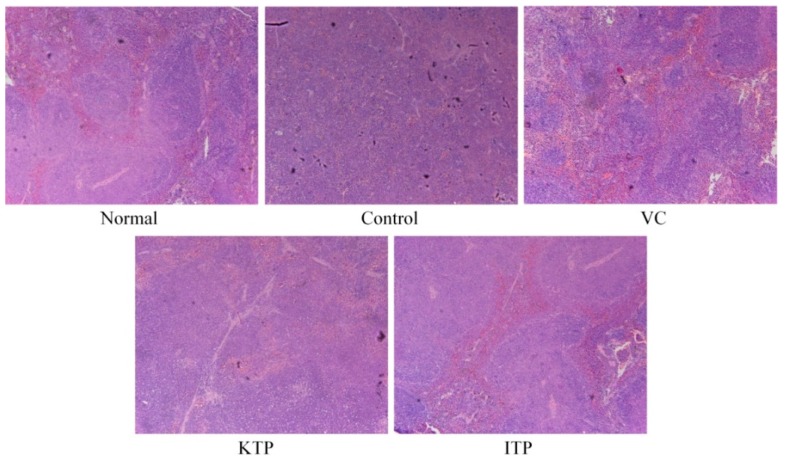
H&E pathological observation of spleen in mice. Magnification 100×. VC: vitamin C (200 mg/kg body weight); KTP: Kuding tea polyphenols (200 mg/kg body weight); ITP: insect tea polyphenols (200 mg/kg body weight).

As shown in Figure 3, the structure of the spleen of mice in the normal group was complete, and the structure of the white and red pulp was clear; the structure of the splenic corpuscle was complete, clear and neatly arranged; and the cells were denser. The white pulp of the spleen in the mice of control group narrowed, the cells decreased, the red pulp was enlarged, and the blood was congested. The red and white pulp of the spleen of the mice in the ITP group, VC group and KTP were demarcated, and the white pulp increased; the lymphatic sheath around the central small artery of the spleen appeared dilated, the lymphocytes were dense, and the red blood sinuses were expanded and congested ITP could show the morphology closest to the normal mice, the effects of VC were also better than KTP.

### 2.4. mRNA and Protein Expression of nNOS, eNOS and iNOS

**Figure 4:**
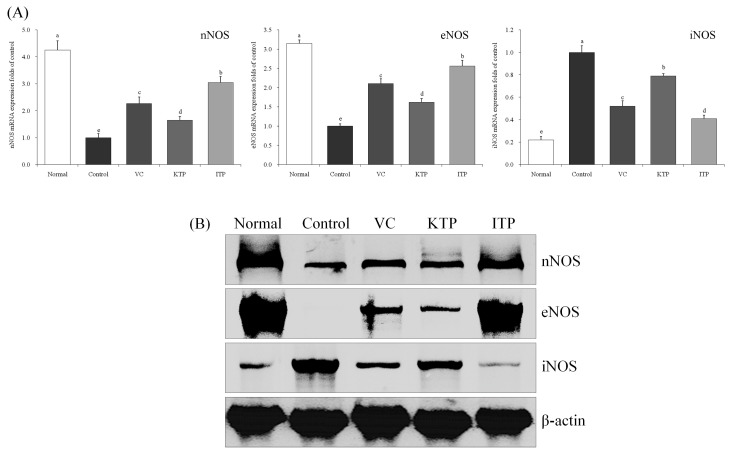
Expression of mRNA (**A**) and protein (**B**) for nNOS, eNOS and iNOS in liver of mice. ^a–e^ Mean values with different letters in the same bar represent significant differences (*p* < 0.05) according to Duncan’s multiple-range test. VC: vitamin C (200 mg/kg body weight); KTP: Kuding tea polyphenols (200 mg/kg body weight); ITP: insect tea polyphenols (200 mg/kg body weight).

**Figure 5:**
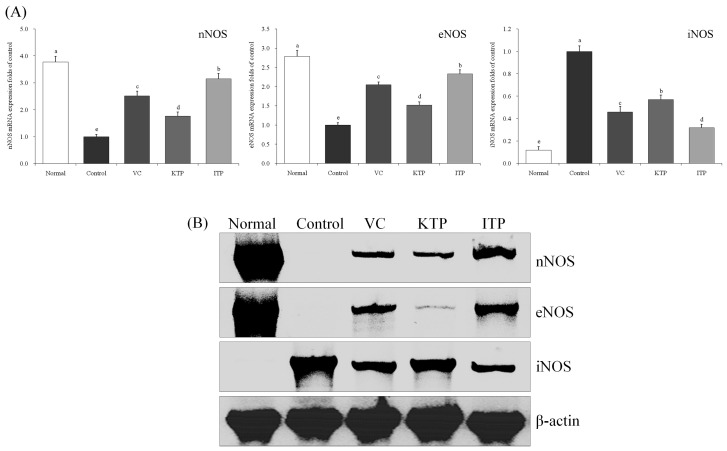
Expression of mRNA (**A**) and protein (**B**) for nNOS, eNOS and iNOS in spleen of mice. ^a–e^ Mean values with different letters in the same bar represent significant differences (*p* < 0.05) according to Duncan’s multiple-range test. VC: vitamin C (200 mg/kg body weight); KTP: Kuding tea polyphenols (200 mg/kg body weight); ITP: insect tea polyphenols (200 mg/kg body weight).

As shown in Figure 4 and Figure 5, the nNOS, eNOS mRNA and protein expression in the liver and spleen tissues of mice in the normal group were strongest and the iNOS expressions were weaker. The mice in the control group had the strongest iNOS expression and weakest nNOS and eNOS expression. ITP, VC and KTP treatment could increase nNOS and eNOS expressions and reduce iNOS expression in liver and spleen tissues compared to mice in the control group, and these effects of ITP were stronger than those of VC and KTP.

### 2.5. mRNA and Protein Expression of Mn-SOD, Cu/Zn-SOD and CAT

**Figure 6:**
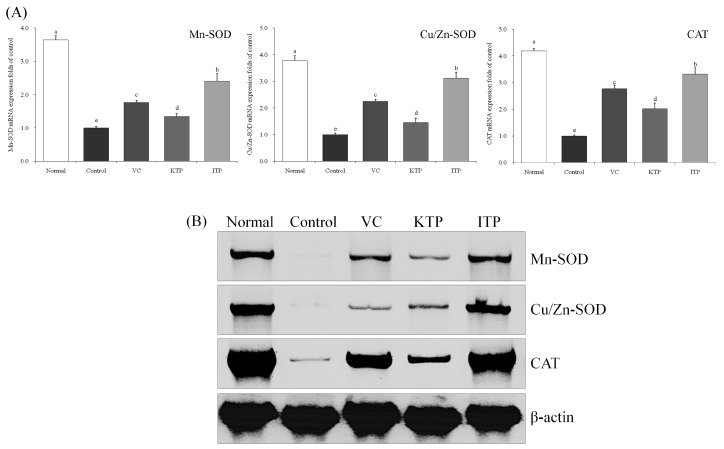
Expression of mRNA (**A**) and protein (**B**) for Mn-SOD, Cu/Zn-SOD and CAT in liver of mice. ^a–e^ Mean values with different letters in the same bar represent significant differences (*p* < 0.05) according to Duncan’s multiple-range test. VC: vitamin C (200 mg/kg body weight); KTP: Kuding tea polyphenols (200 mg/kg body weight); ITP: insect tea polyphenols (200 mg/kg body weight).

**Figure 7:**
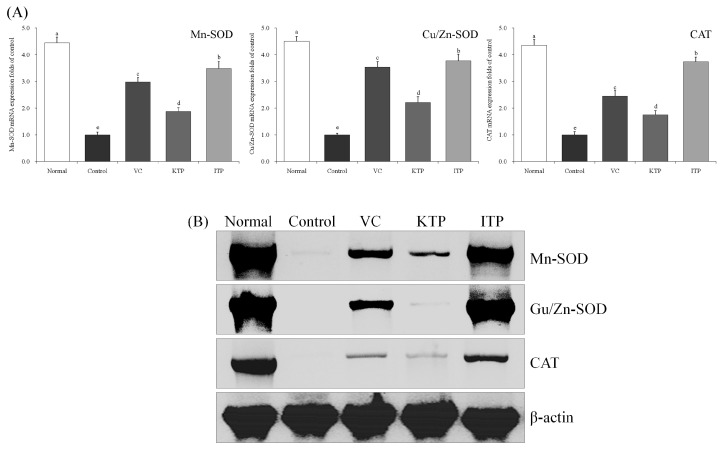
Expression of mRNA (**A**) and protein (**B**) for Mn-SOD, Cu/Zn-SOD and CAT in spleen of mice. ^a–e^ Mean values with different letters in the same bar represent significant differences (*p* < 0.05) according to Duncan’s multiple-range test. VC: vitamin C (200 mg/kg body weight); KTP: Kuding tea polyphenols (200 mg/kg body weight); ITP: insect tea polyphenols (200 mg/kg body weight).

As shown in Figure 6 and Figure 7, the mRNA and protein expression of Mn-SOD, Cu/Zn-SOD and CAT in the liver and spleen tissues of mice in control group were weakest, but these expressions in mice from the control group were strongest. ITP, VC and KTP treatment could increase this expression compared to the control group, the expression of ITP-treated mice was stronger than for VC- and KTP-treated mice, and VC treated mice also had stronger expression than KTP-treated mice.

### 2.6. mRNA and Protein Expression of HO-1 and Nrf2

**Figure 8:**
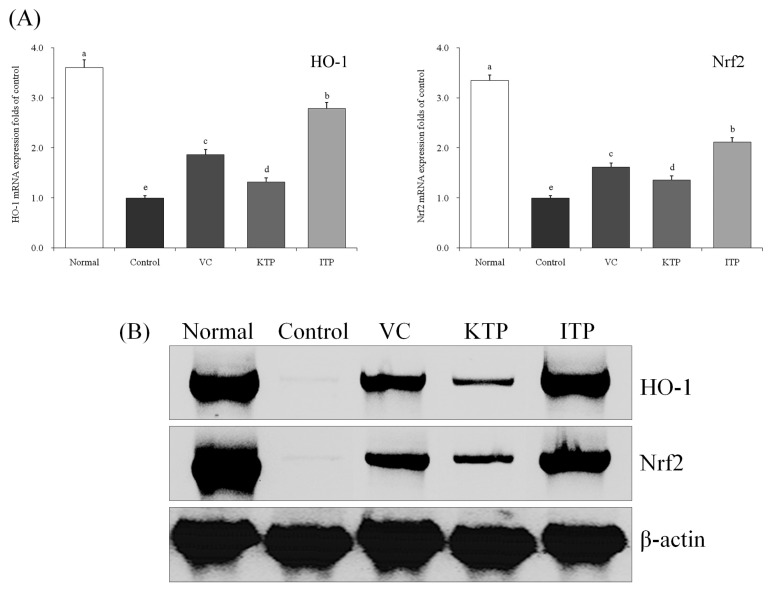
Expression of mRNA (**A**) and protein (**B**) for HO-1 and Nrf2 in liver of mice. ^a–e^ Mean values with different letters in the same bar represent significant differences (*p* < 0.05) according to Duncan’s multiple-range test. VC: vitamin C (200 mg/kg body weight); KTP: Kuding tea polyphenols (200 mg/kg body weight); ITP: insect tea polyphenols (200 mg/kg body weight).

**Figure 9:**
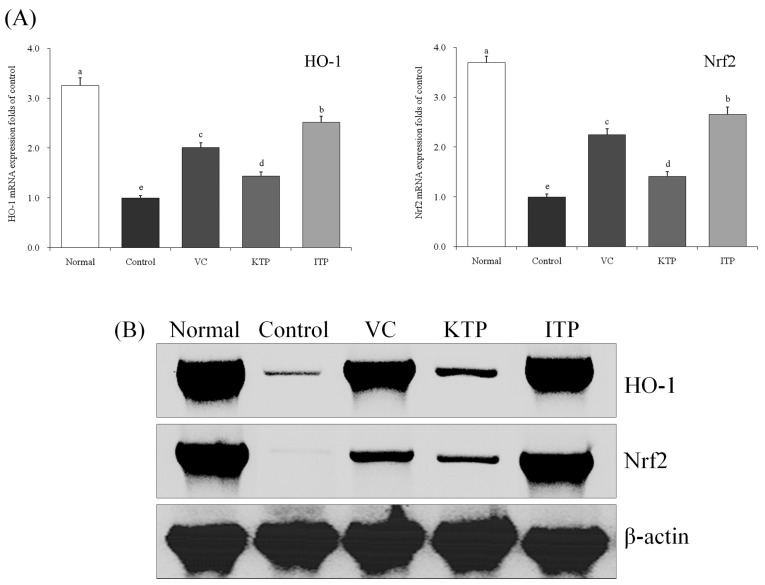
Expression of mRNA (**A**) and protein (**B**) for HO-1 and Nrf2 in spleen of mice. ^a–e^ Mean values with different letters in the same bar represent significant differences (*p* < 0.05) according to Duncan’s multiple-range test. VC: vitamin C (200 mg/kg body weight); KTP: Kuding tea polyphenols (200 mg/kg body weight); ITP: insect tea polyphenols (200 mg/kg body weight).

As shown in Figure 8 and Figure 9, the normal group mice had stronger HO-1 and Nrf2 mRNA and protein expression in liver and spleen tissues than other groups. The HO-1 and Nrf2 expressions of ITP group mice were only weaker than the normal group mice; the VC group mice also had stronger HO-1 and Nrf2 expression than the KTP and control groups.

### 2.7. Expression of mRNA and Protein for γ-GCS and NQO1

**Figure 10:**
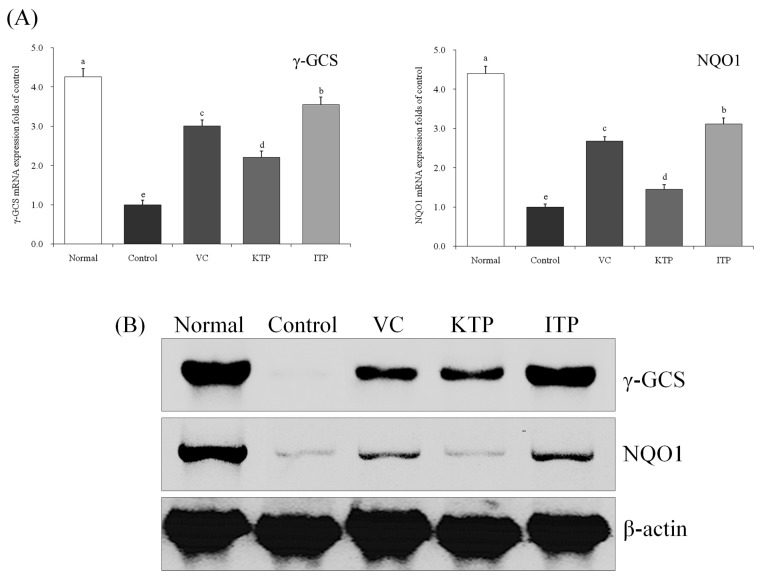
Expression of mRNA (**A**) and protein (**B**) for γ-GCS and NQO1 in liver of mice. ^a–e^ Mean values with different letters in the same bar represent significant differences (*p* < 0.05) according to Duncan’s multiple-range test. VC: vitamin C (200 mg/kg body weight); KTP: Kuding tea polyphenols (200 mg/kg body weight); ITP: insect tea polyphenols (200 mg/kg body weight).

**Figure 11:**
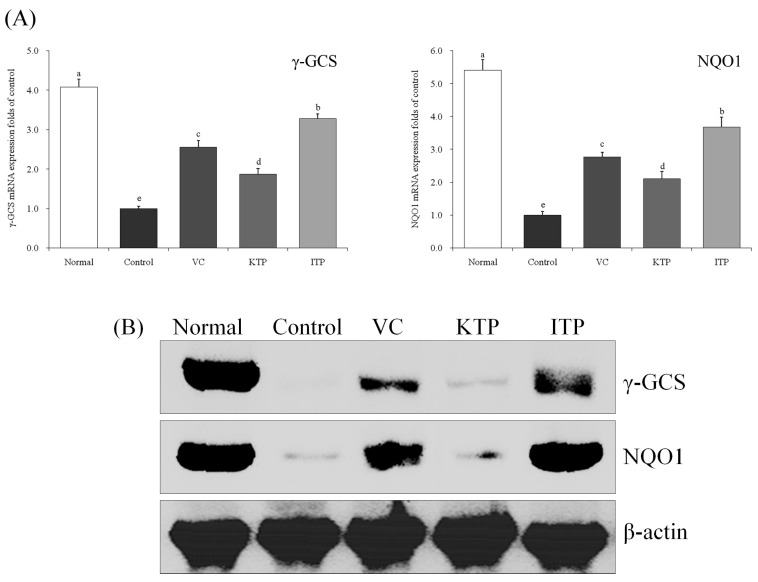
Expression of mRNA (**A**) and protein (**B**) for γ-GCS and NQO1 in spleen of mice. ^a–e^ Mean values with different letters in the same bar represent significant differences (*p* < 0.05) according to Duncan’s multiple-range test. VC: vitamin C (200 mg/kg body weight); KTP: Kuding tea polyphenols (200 mg/kg body weight); ITP: insect tea polyphenols (200 mg/kg body weight).

As shown in Figure 10 and Figure 11, the mRNA and protein expression of γ-GCS and NQO1 in the liver and spleen of mice in the normal group were strongest, but the expressions of mice in the control group was weakest. After treatment with ITP, VC and KTP, the γ-GCS and NQO1 expression increased, and the expression in ITP-treated mice was stronger than for V- and KTP-treated mice; only KTP-treated mice had γ-GCS and NQO1 expression stronger than control group mice.

## 3. Discussion

Tea polyphenols can form precipitates with metal ions, and thus, tea polyphenols can be separated from tea extracts to obtain higher-purity tea polyphenols. Ion precipitation solvent extraction is often used for Kuding tea and Insect tea extraction. Hydrochloric acid is used to dissolve polyphenols precipitated with ions, and in our other experiments (not published), Kuding tea (raw tea of Insect tea) polyphenols contained chlorogenic acid, cryptochlorogenic acid, and isochlorogenic acids A, B, and C. Various extraction methods have been compared by other scholars, but the purity of polyphenols extracted by this method is the highest, and precipitants and hydrochloric acid have no effect on the composition. Thus, this extraction method was used in this study.

Hydroxytyrosol is a polyphenol with antioxidant response, and hydroxytyrosol can activate the transcription factors of PPAR-α and Nrf2, through NF-κB activation reducing; hydroxytyrosol inhibits HFD-induced liver alteration [20]. Polyphenols can also prevent metabolic alterations in desaturase activities and oxidative stress in liver-injured mice [21]. Polyphenols have antioxidant effects, not only in chemical oxidation but also for high fat factors.

Nitric oxide (NO) is produced through the oxidation of l-arginine by nitric oxide synthase (NOS). Because NO has a very short half-life, most studies of NO function are based on the regulation of NOS activity [22]. Currently, there are three types of NOS involved in the normal physiological or pathological process of NO: nNOS, eNOS and iNOS [23]. Nitric oxide is a typical free radical with an unpaired electron on the nitrogen atom. NO has a strong oxidizability and can pass through the cell membrane freely. Under physiological conditions, it commonly acts on soluble guanylate cyclase acid and plays a role in its biology, but in the case of excessive NO, it coordinates with O_2_^−^ synergistically to cause cell damage [24]. NO can form ONOO^−^ with a superoxide anion, inactivate Mn-SOD in mitochondria, generate numerous free radicals and mediate oxidative damage. Under physiological conditions, nNOS and eNOS can be expressed (but iNOS cannot), and the distribution of nNOS is most extensive, mainly located in the central neurons, especially the cerebellar granule cell layer and molecular layer [25]. Down-regulation of nNOS protein can inhibit the activity of cultured cerebellar granule cells in vitro and reduce the survival rate of cerebellar granulosa cells; nNOS can promote the survival of mature cerebellar granulosa cells and may be related to ageing [26]. In addition, eNOS can activate soluble guanylate cyclase in vascular smooth muscle cells to increase the concentration of cAMP intracellularly, which can dilate blood vessels, inhibit platelet and leukocyte adhesion and aggregation and have a neuroprotective function [27]. iNOS is mainly distributed into macrophages, inflammatory neutrophils, vascular smooth muscle cells, endothelial cells, microglia and astrocytes. Once synthesized, NO is continuously produced until the substrate is depleted, and the excess NO can damage the tissue through active nitrogen and oxidative stress effects [28]. In this study, ITP and KTP could reduce NO and iNOS levels and increase nNOS and eNOS levels in the body of oxidatively damage mice; NO, nNOS, eNOS and iNOS are the factors of oxidative damage, so maintaining these factors at normal levels could inhibit oxidative damage [25,26,27,28]. ITP and KTP may have reduced the oxidative damage through the nitric oxide pathway, and ITP had stronger effects than KTP. 

SOD is an important antioxidant enzyme in organisms, and it is widely distributed in various organisms, such as animals, plants and microorganisms. SOD has a special physiological activity and its primary role is to remove free radicals from an organism. The level of SOD in the body is an intuitive indicator of ageing and death; it has been confirmed that as many as 60 diseases are caused by oxygen free radicals [29]. SOD can antagonize and block the damage caused by oxygen free radicals to cells, repair damaged cells in time, and restore the cell damage caused by free radicals. Due to the pressures of modern life, including environmental pollution, radiation and excessive exercise, numerous oxygen free radicals are formed, and thus, the role of SOD in biological antioxidant mechanisms is becoming increasingly significant. O_2_^−^ is mainly produced during the process of oxygen consumption and has a lively chemical property. Low concentrations of oxygen free radicals are particularly important for maintaining normal life activities, but when the concentration is too high, it can destroy the body’s macromolecular protein structure and lead to metabolic disorders in tissue cells [30]. SOD has an antioxidant capacity and plays an important role in the defense of oxidative stress. It can catalyze the disproportionation reaction of O_2_^−^ and convert harmful O_2_^−^ into hydrogen peroxide. Although hydrogen peroxide is still reactive oxygen that is harmful to the body, CAT and POD in the body can break it down into completely harmless water immediately [31]. Thus, the enzymes form a complete antioxidation chain, avoiding damage of the reactive oxygen to tissue cells. SOD in animals mainly exists in blood cytosolic CuZn-SOD, and Mn-SOD distributes in the mitochondrial matrix [32]. Mn-SOD is considered a key barrier against mitochondrial oxidative damage. With increased bodily oxygen consumption, significant oxygen free radicals will be generated, which will lead to a strong inducement of Mn-SOD expression. If the production of O_2_^−^ is far greater than elimination from the body, it can cause an imbalance between oxidation and antioxidation, eventually leading to oxidative damage and ageing [33]. Studies have shown that some atrophic tissue diseases can lead to decreased expression of Mn-SOD; atherosclerosis can also decrease the expression of Cu/Zn-SOD and Mn-SOD mRNA in blood vessels. In the presence of oxidative damage and chemical induction of ageing, the content of Cu/Zn-SOD in the body will also be significantly reduced [34,35]. Increasing SOD activity can inhibit oxidative damage, raising CuZn-SOD and Mn-SOD activities. Tea polyphenols could reduce oxidative damage by increasing SOD, CuZn-SOD and Mn-SOD activities [10,31,32,33]. From the results of this study, we found that ITP-treated mice had stronger SOD (contained Cu/Zn-SOD and Mn-SOD) activities in serum and tissue than KTP-treated mice. 

The main function of GSH-Px is to eliminate lipid hydroperoxide. The large amount of GSH-Px in the tissue can remove H_2_O_2_ rapidly [36]. In pathological and physiological conditions, reactive oxygen species such as ·OH may induce lipid peroxidation. In addition to causing direct damage to the biofilm, GSH-Px may also react with protein and nucleic acid through lipid hydroperoxide to causes extensive damage to the body [37]. Lipid peroxidation is also one reason for cell ageing. Preventing lipid peroxidation can delay cell ageing, so GSH-Px plays an important role in preventing oxidation and ageing [38]. Therefore, measurement of MDA can often reflect the degree of lipid peroxidation in the body, which indirectly reflects the extent of cellular damage [39,40]. GSH-Px and MDA are also important factors related to oxidative damage, and raising GSH-Px and reducing MDA could decrease oxidative damage in mice. ITP treatment could also reduce oxidative damage by increasing GSH-Px activity and reducing MDA activities, and the antioxidative effects of ITP were better than VC and KTP treatments.

HO-1 is an important antioxidant enzyme that plays a key role in the protection of endogenous and exogenous origin cells from harmful stimuli. Its antioxidant function is related to the prevention of free hemoglobin in oxidation. HO-1 and its enzyme products such as bilirubin and CO play an important role in antioxidation, anti-inflammation, inhibition of apoptosis, vasodilation, and improvement of tissue microcirculation [41,42]. Under normal physiological conditions, HO-1 expression and vitality is low, but under stress conditions, such as hemoglobin, hydrogen peroxide (H_2_O_2_), endotoxin, heavy metals, ultraviolet radiation, or high/low oxygen, the expression of HO-1 through Nrf2 can show an obvious antioxidant effect [43]. The Nrf2/HO-1 pathway is widely involved in the antioxidative stress injury of tissues and organs such as heart, liver, kidney and the nervous system; it is one of the most important endogenous protection systems [44]. Studies have found that pigment glycosides up-regulate HO-1 scavenge ROS through the serine/threonine protein kinase (Akt) and extracellular regulatory protein kinase (ERK1/2)/Nrf2 signaling pathway, which plays a protective role in hepatotoxicity [45,46]. In addition, other studies have shown that some of the active ingredients in natural plants prevent oxidative stress and reduce cytotoxicity through the Nrf2/HO-1 pathway [47,48], which is related oxidative damage. Low Nrf2 and HO-1 levels could increase oxidative damage, and inhibiting the Nrf2 and HO-1 levels could keep the body healthy [41,43,48]. ITP as a functional material showed antioxidative effects through the Nrf2/HO-1 pathway, and these effects were higher than for VC and KTP.

GSH is a highly efficient oxygen free radical/nitrogen radical scavenger and an important factor to maintain cellular redox status and homeostasis. It is also involved in immune regulation, remodeling of the intracellular and extracellular matrix, apoptosis and mitochondrial respiration and is used as an intermediate to store and transfer cysteine. As an antioxidant, it reacts with free radicals to become oxidized to protect tissues and cells from free radical attack and damage [49]. γ-GCS is the rate-limiting enzyme synthesized by GSH, which determines the level of GSH in vivo and is an important antioxidant factor [50]. At the same time, Nrf2 binding sequence is an enhancer of γ-GCS that is regulated by Nrf2. Nrf2 gene knockdown can decrease expression of the two subunits of γ-GCS in mouse fibroblasts and hepatocytes. Nrf2 and γ-GCS mRNA are highly correlated to the protein [51]. NQO1 is a protective reductase in cells that protects them from quinones and oxidative damage. However, the reduction products of NQO1 may be further metabolized or oxidized under different oxygen environments, showing different effects. NQO1 is a downstream gene of Nrf2, and its expression will be increased and play a protective role in cells when Nrf2 is activated [52]. γ-GCS and NQO1 had effects on oxidative damage, and high levels of γ-GCS and NQO1 could help to reduce oxidative damage [50,51,52]. In this study, ITP also had increasing effects on γ-GCS and NQO1 expression, and these effects could contribute to antioxidation; the effects were stronger than those of VC and KTP.

## 4. Materials and Methods

### 4.1. Polyphenol Extraction

The production of Kuding tea is similar to the production of green tea. Fresh Kuding tea is made through withering, fixing, rolling and drying. Kuding tea is also the raw tea of insect tea, for which the Kuding tea leaves are stacked in a bucket. The layers are evenly covered with rice water, covered and kept moist, and the leaves gradually ferment and rot. Insect tea is made of insect larvae faces following the insects’ consumption of Kuding tea. The 100 g samples (Kuding tea and Insect tea from local market, Guizhou, China) were crushed: the insect tea was crushed from the main origin, the Kuding tea was the raw tea of insect tea, and both were used in this study. The Kuding tea and insect tea were added to 100 mL of a 45% (volume ratio) ethanol solution, soaked for 30 min at 90 °C, and soaked one more time. The two liquid fractions were mixed and liquid’s pH was adjusted to 6.0. Then, 160 mL of mixed precipitant of AlCl_3_ (6 g) and ZnCl_2_ (12 g) was added to the precipitate, and the mixture was centrifuged (3000 r/min, 10 min); 200 mL of hydrochloric acid (12% by volume) was added to the collected precipitate in the transfer, the supernatant was separated, and 200 mL ethyl acetate was added for extraction. Finally, the extracted liquid was rotationally evaporated to give polyphenol extracts. Chlorogenic acid was used as a standard, and the polyphenol contents of KTP and ITP were determined using ultraviolet spectrophotometry [53]. 

### 4.2. Polyphenols Contents Determination

The chlorogenic acid was dissolved in distilled water to prepare different concentrations of chlorogenic acid solution. According to the Folin-Ciocalteu colorimetric method, 3 mL of Folin-Ciocalteu chromogenic agent and 4.5 mL saturated Na_2_CO_3_ solution were added to the chlorogenic acid solution (1 mL) at different concentrations and diluted to a constant volume to 25 mL. The absorbance of the solution after color rendering was measured at 747 nm. The absorbance was the X-axis, and the concentration of chlorogenic acid solution was the Y-axis coordinate to construct a standard concentration curve for chlorogenic acid. KTP and ITP were dissolved in distilled water to determine the content of polyphenols, and the above method was used to determine the polyphenol content. 

### 4.3. Animal Experiments

Fifty male Kunming (KM) mice (7 weeks old, male, body weight: 24.47 ± 0.32 g) were purchased from the Experimental Animal Center of Chongqing Medical University (Chongqing, China). The KM mice were fed in a temperature- (25 ± 2 °C) and humidity- (50 ± 5%) controlled facility with a 12-h light/dark cycle and free access to a standard diet and water. The KM mice were divided into five groups: normal group, control group, vitamin C (VC) group, Kuding tea polyphenols (KTP) group and insect tea polyphenols (ITP) group. During the experiment, the mice in the normal group were only treated with distilled water (0.2 mL) by gavage every day. In the first 2 weeks of the experiment, the mice in the control group were also only treated with distilled water (0.2 mL) by gavage every day; the mice in the VC group, KTP group and ITP group were treated with 200 mg/kg b.w. of vitamin C, KTP or ITP every day, respectively. The mice in groups except the normal group were treated with d-galactose (120 mg/kg b.w.) by intraperitoneal injection for 6 weeks, and mice in all groups were treated with this treatment in the first 2 weeks. After 8 weeks, all mice were killed using the broken neck method, and the plasma, liver and spleen of mice were collected [54]. The present study was approved by the Animal Ethics Committee of Chongqing University of Education (SYXK (Yu) 2017-0001).

### 4.4. Determination of Activities of NO, SOD, GSH-Px and MDA

The activities of NO, SOD, GSH-Px and MDA in serum and tissue were determined using kits for NO (A012-1, Nanjing Jiancheng Bioengineering Institute, Nanjing, Jiangsu, China), SOD (A001-3), GSH-Px (A005), GSH (A006-1) and MDA (A003-1).

### 4.5. Pathological Observation

The liver and spleen from mice were placed in a 10% formalin solution for 24 h and dehydrated in 95% ethanol for 24. The tissues were cut into hematoxylin and eosin (H&E) sections for observation, and the sections were observed using a BX43 microscope (Olympus, Tokyo, Japan).

### 4.6. Total RNA Extraction and Quantitative Real-Time Polymerase Chain Reaction (qRT-PCR) Assay

The homogenate from liver and spleen tissues was collected, and the total RNA was extracted using TRIzol reagent (Thermo Fisher Scientific, Waltham, MA, USA). The concentration of RNA was detected by micro-UV spectrophotometer (Nano 300, Aosheng, Hanzhou, Zhejiang, China), and 1 μg mRNA was reverse transcribed into cDNA. PCR reaction program: pre-denaturation for 3 min at 95 °C, denaturation for 10 s at 95 °C, annealing for 30 s at 57 °C, extension for 15 s at 72 °C, 40 cycles. The primers in this study are shown in Table 5. The relative transcription levels of the mRNAs were calculated according to the 2^−^^ΔΔ^^Cr^ formula [55].

### 4.7. Protein Extraction and Western Blot Analysis

The homogenate from the liver tissue was collected, and the lysate was used to lyse the hepatocytes for 30 min on ice. The BCA method was used to determine the protein concentration. The sample was added to 5× loading buffer by volume and heated in a water bath at 100 °C for 5 min. The hepatocytes were sonicated by SJIALAB for 2 min (10% ultrasound intensity) and centrifuged for 10 min to obtain the supernatant. The extracted protein was subjected to polyacrylamide gel electrophoresis (80–120 V), transferred to a PVDF membrane, sealed and incubated overnight at 4 °C with the addition of primary antibody of nNOS (3G6B10, Invitrogen, Thermo Fisher Scientific, Waltham, MA, USA), eNOS (9D10, Invitrogen), iNOS (PA1036, Invitrogen), NF-κB (PA1186, Invitrogen), IκB-α (397700, Invitrogen), Mn-SOD (PA530604, Invitrogen), Cu/Zn-SOD (PA5270240, Invitrogen), CAT (PA259183, Invitrogen), HO-1 (ab68477, Abcam, Cambridge, MA, USA), Nrf2 (ab92946, Abcam), γ-GCS (sc-390811, Santa Cruz Biotechnology, Dallas, TX, USA), NQO1 (ab28947, Abcam) and β-action (MA5157739, Invitrogen). The protein was incubated overnight at 37 °C with the addition of a second antibody (A21241) for 2 h, and color detection and chemiluminescence imaging analysis system (Tanon 6200, ShanghaiTanon Technology Co., Ltd., Shanghai, China) were used to collect images [56].

### 4.8. Statistical Analysis

The experimental data was presented as the mean ± standard deviation. Differences between the mean values for each group were calculated by one-way ANOVA with Duncan’s multiple range tests. *p* < 0.05 was considered a statistically significant difference. The SAS v9.1 statistical software package (SAS Institute Inc., Cary, NC, USA) was used for analysis.

## 5. Conclusions

**Figure 12:**
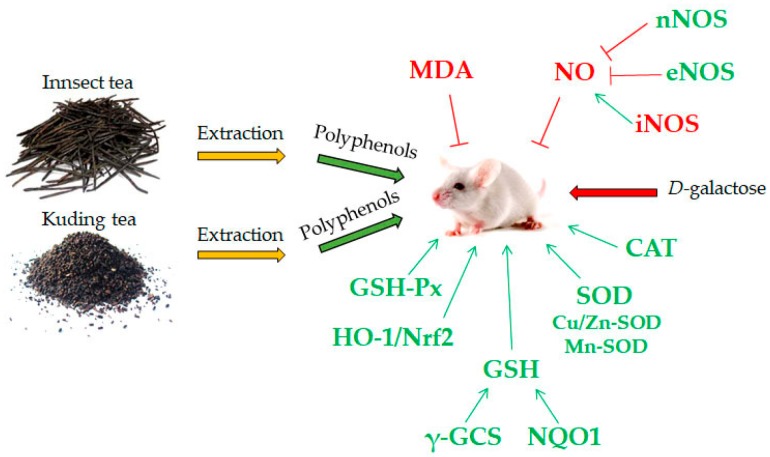
The observed effects in this study.

In this study, we used the d-galactose-induced oxidation mouse model to determine the antioxidative effects of the polyphenols of insect tea and its raw tea (Kuding tea). ITP increased SOD, GSH-Px, GSH activities and decreased the NO, MDA activities in the serum, liver and spleen of mice compared to oxidation mice (control group); the effects of ITP were higher than for VC and KTP. The pathological observation also showed that ITP, VC and KTP could reduce the oxidative damage to liver and spleen tissues, and the effects of ITP were the strongest among the three. The mRNA and protein expression of nNOS, eNOS, Mn-SOD, Cu/Zn-SOD, CAT, HO-1, Nrf2, γ-GCS, NQO1 in the liver and spleen tissues of mice in the ITP group were stronger than those for mice in the VC and KTP group, but iNOS expression of mice in the ITP group were weaker than for mice in the VC and KTP group. From these results (Figure 12), we conclude that the antioxidative effects of ITP were better than for KTP, and the transformation of insects could improve the antioxidant effect of KTP. ITP could be used as a raw material in an antioxidant and anti-ageing functional food.

## Figures and Tables

**Table 1:** Polyphenol contents of KTP and ITP.

Group	OD_747_ Value	Polyphenols (μg)	Polyphenol Extract (μg)	Polyphenols Content (%)
KTP	0.172 ± 0.03	35.69 ± 1.02	50.00 ± 0.00	71.38 ± 1.06
ITP	0.168 ± 0.04	34.91 ± 0.82	50.00 ± 0.00	69.82 ± 1.11

Values presented are mean ± standard deviation. KTP: Kuding tea polyphenols; ITP: Insect tea polyphenols.

**Table 2:** Activities of NO, SOD, GSH-Px, GSH and MDA in serum of mice.

Group	NO (μmol/L)	SOD (U/mL)	GSH-Px (U/mL)	GSH (mg/L)	MDA (nmol/mL)
Normal	22.52 ± 1.08 ^e^	267.52 ± 9.45 ^a^	169.12 ± 4.32 ^a^	31.21 ± 2.71 ^a^	7.08 ± 0.42 ^e^
Control	69.71 ± 2.21 ^a^	122.35 ± 6.71 ^e^	101.20 ± 2.08 ^e^	14.35 ± 1.89 ^e^	25.12 ± 1.32 ^a^
VC	40.52 ± 1.56 ^c^	174.50 ± 7.46 ^c^	133.25 ± 2.15 ^c^	20.25 ± 1.75 ^c^	13.12 ± 0.62 ^c^
KTP	50.85 ± 1.44 ^b^	155.69 ± 6.03 ^d^	118.74 ± 2.20 ^d^	17.35 ± 1.26 ^d^	17.42 ± 0.49 ^b^
ITP	36.12 ± 1.32 ^d^	208.64 ± 6.42 ^b^	149.45 ± 2.33 ^b^	25.33 ± 2.03 ^b^	9.36 ± 0.43 ^d^

Values presented are mean ± standard deviation (*N* = 10/group). ^a–e^ Mean values with different letters over same column represent significant differences (*p* < 0.05) according to Duncan’s multiple range test. VC: vitamin C (200 mg/kg body weight); KTP: Kuding tea polyphenols (200 mg/kg body weight); ITP: Insect tea polyphenols (200 mg/kg body weight).

**Table 3:** Activities of NO, SOD, GSH-Px, GSH and MDA in liver of mice.

Group	NO (μmol/gprot)	SOD (U/mgprot)	GSH-Px (U/mgprot)	GSH (mg/ gprot)	MDA (nmol/mgprot)
Normal	3.85 ± 0.19 ^e^	98.78 ± 6.20 ^a^	178.92 ± 5.69 ^a^	7.33 ± 0.41 ^a^	2.36 ± 0.12 ^e^
Control	9.78 ± 0.39 ^a^	36.52 ± 2.39 ^e^	88.15 ± 3.56 ^e^	2.58 ± 0.25 ^e^	8.87 ± 0.35 ^a^
VC	6.32 ± 0.23 ^c^	62.12 ± 3.85 ^c^	126.20 ± 3.98 ^d^	5.10 ± 0.33 ^d^	5.06 ± 0.30 ^c^
KTP	7.71 ± 0.18 ^b^	45.12 ± 2.48 ^d^	111.25 ± 3.25 ^c^	4.04 ± 0.19 ^c^	6.12 ± 0.24 ^b^
ITP	5.05 ± 0.22 ^d^	79.82 ± 4.75 ^b^	145.12 ± 4.56 ^b^	6.21 ± 0.22 ^b^	4.23 ± 0.17 ^d^

Values presented are mean ± standard deviation (*N* = 10/group). ^a–e^ Mean values with different letters over same column represent significant differences (*p* < 0.05) according to Duncan’s multiple range test. VC: vitamin C (200 mg/kg body weight); KTP: Kuding tea polyphenols (200 mg/kg body weight); ITP: Insect tea polyphenols (200 mg/kg body weight).

**Table 4:** Activities of NO, SOD, GSH-Px, GSH and MDA in spleen of mice.

Group	NO (μmol/gprot)	SOD (U/mgprot)	GSH-Px (U/mgprot)	GSH (mg/ gprot)	MDA (nmol/mgprot)
Normal	1.87 ± 0.12 ^e^	63.15 ± 3.75 ^a^	107.23 ± 4.56 ^a^	5.41 ± 0.24 ^a^	1.12 ± 0.08 ^e^
Control	6.32 ± 0.23 ^a^	21.65 ± 2.21 ^e^	42.18 ± 3.21 ^e^	1.79 ± 0.14 ^e^	4.32 ± 0.12 ^a^
VC	3.69 ± 0.22 ^c^	40.58 ± 2.11 ^c^	77.12 ± 2.42 ^d^	3.87 ± 0.20 ^d^	2.23 ± 0.14 ^c^
KTP	4.41 ± 0.19 ^b^	32.01 ± 2.33 ^d^	62.10 ± 3.87 ^c^	3.11 ± 0.16 ^c^	2.79 ± 0.09 ^b^
ITP	2.48 ± 0.21 ^d^	52.17 ± 2.36 ^b^	88.17 ± 4.02 ^b^	4.42 ± 0.21 ^b^	1.87 ± 0.10 ^d^

Values presented are mean ± standard deviation (*N* = 10/group). ^a–e^ Mean values with different letters over same column represent significant differences (*p* < 0.05) according to Duncan’s multiple range test. VC: vitamin C (200 mg/kg body weight); KTP: Kuding tea polyphenols (200 mg/kg body weight); ITP: Insect tea polyphenols (200 mg/kg body weight).

**Table 5:** Sequences of qPCR primers used in this study.

Gene Name	Sequence
nNOS	Forward: 5′-ACGGCAAACTGCACAAAGC-3′
	Reverse: 5′-CGTTCTCTGAATACGGGTTGTTG-3′
eNOS	Forward: 5′-TCAGCCATCACAGTGTTCCC-3′
	Reverse: 5′-ATAGCCCGCATAGCGTATCAG-3′
iNOS	Forward: 5′-GTTCTCAGCCCAACAATACAAGA-3′
	Reverse: 5′-GTGGACGGGTCGATGTCAC-3′
Mn-SOD	Forward: 5′-CAGACCTGCCTTACGACTATGG-3′
	Reverse: 5′-CTCGGTGGCGTTGAGATTGTT-3′
Gu/Zn-SOD	Forward: 5′-AACCAGTTGTGTTGTCAGGAC-3′
	Reverse: 5′-CCACCATGTTTCTTAGAGTGAGG-3′
CAT	Forward: 5′- GGAGGCGGGAACCCAATAG-3′
	Reverse: 5′- GTGTGCCATCTCGTCAGTGAA-3′
HO-1	Forward: 5′-ACAGATGGCGTCACTTCG-3′
	Reverse: 5′-TGAGGACCCACTGGAGGA-3′
Nrf2	Forward: 5′-CAGTGCTCCTATGCGTGAA-3′
	Reverse: 5′-GCGGCTTGAATGTTTGTC-3′
γ-GCS	Forward: 5′-GCACATCTACCACGCAGTCA-3′
	Reverse: 5′-CAGAGTCTCAAGAACATCGCC-3′
NQO1	Forward: 5′-CTTTAGGGTCGTCTTGGC-3′
	Reverse: 5′-CAATCAGGGCTCTTCTCG-3′
GAPDH	Forward: 5′-AGGTCGGTGTGAACGGATTTG-3′
	Reverse: 5′-GGGGTCGTTGATGGCAACA-3′
